# High Diversity and Variability in the Vaginal Microbiome in Women following Preterm Premature Rupture of Membranes (PPROM): A Prospective Cohort Study

**DOI:** 10.1371/journal.pone.0166794

**Published:** 2016-11-18

**Authors:** Teenus Paramel Jayaprakash, Emily C. Wagner, Julie van Schalkwyk, Arianne Y. K. Albert, Janet E. Hill, Deborah M. Money, PPROM Study Group

**Affiliations:** 1 Department of Veterinary Microbiology, University of Saskatchewan, Saskatoon, SK, Canada; 2 Women’s Health Research Institute, BC Women’s Hospital and Health Centre, Vancouver, BC, Canada; 3 Department of Obstetrics and Gynaecology, University of British Columbia, Vancouver, BC, Canada; National Institute of Health, ITALY

## Abstract

**Objective:**

To characterize the vaginal microbiota of women following preterm premature rupture of membranes (PPROM), and determine if microbiome composition predicts latency duration and perinatal outcomes.

**Design:**

A prospective cohort study

**Setting:**

Canada

**Population:**

Women with PPROM between 24^+0^ and 33^+6^ weeks gestational age (GA).

**Methods:**

Microbiome profiles, based on pyrosequencing of the *cpn*60 universal target, were generated from vaginal samples at time of presentation with PPROM, weekly thereafter, and at delivery.

**Main Outcome Measures:**

Vaginal microbiome composition, latency duration, gestational age at delivery, perinatal outcomes.

**Results:**

Microbiome profiles were generated from 70 samples from 36 women. Mean GA at PPROM was 28.8 wk (mean latency 2.7 wk). Microbiome profiles were highly diverse but sequences representing *Megasphaera* type 1 and *Prevotella* spp. were detected in all vaginal samples. Only 13/70 samples were dominated by *Lactobacillus* spp. Microbiome profiles at the time of membrane rupture did not cluster by gestational age at PPROM, latency duration, presence of chorioamnionitis or by infant outcomes. *Mycoplasma* and/or *Ureaplasma* were detected by PCR in 81% (29/36) of women, and these women had significantly lower GA at delivery and correspondingly lower birth weight infants than *Mycoplasma* and/or *Ureaplasma* negative women.

**Conclusion:**

Women with PPROM had mixed, abnormal vaginal microbiota but the microbiome profile at PPROM did not correlate with latency duration. *Prevotella* spp. and *Megasphaera* type I were ubiquitous. The presence of Mollicutes in the vaginal microbiome was associated with lower GA at delivery. The microbiome was remarkably unstable during the latency period.

## Introduction

Preterm delivery is the most important contributor to neonatal morbidity and mortality worldwide [1]. Preterm premature rupture of membranes (PPROM) is a precursor to 20–30% of preterm deliveries [2, 3]. If PPROM is remote from term, prolongation of gestation is desirable if no signs of infection and fetal well-being *in utero* can be assured. The latency period between membrane rupture and delivery is a critical period for determining maternal and neonatal health outcomes, as although longer latencies have been associated with reduced odds of neonatal morbidity [4], there is an associated increased risk of maternal and/or fetal infection [5, 6]. This dilemma in clinical management becomes more profound closer to term and results in much debate about optimal management [7]. It is vital to understand whether the composition of the vaginal microbiome would predict safe latency or if particular dysbiosis would predict adverse outcomes for the infant and/or mother.

Abnormal vaginal microbiota is a recognized risk factor for PPROM [8–10], as endogenous bacteria present in the lower genital tract can ascend into the uterus causing an inflammatory response, which can lead to PPROM and/or preterm labour [2, 11]. Historically, anaerobes, Gram negative organisms and mycoplasmas have been associated with intrauterine infection [2, 12, 13], and some specific microbial species including genital *Mycoplasma* spp., *Ureaplasma* spp. [14], and the agents of syphilis, gonorrhea and trichomoniasis have been associated with preterm birth [2]., previous investigations have been greatly limited by dependence on the challenges of culture and isolation of fastidious organisms.

Studies exploiting high throughput DNA sequencing to detect microbes in fetal membranes, placenta and amniotic fluid of women with preterm birth (PTB), PPROM and term labour have resulted in the detection of a wider diversity of microbial species than culture dependent studies and identification of previously unrecognized species [15–18]. These results further support an association between bacterial colonization of fetal membranes and PTB. Recent studies employing sequence-based techniques have shown varied results. Romero *et al*. [19] detected no difference in the microbial profiles of women who subsequently delivered term or preterm, whereas others showed differences in prevalence and abundance of organisms such as *Leptotrichia/Sneathia*, BVAB (bacterial vaginosis-associated bacteria), *Mobiluncus* spp. and *Mycoplasma* spp. in the vaginal microbiomes of women who deliver preterm [20, 21]. To date there has been no comprehensive study, utilizing high-throughput gene sequencing based methodologies, to characterize the microbiome in the context of preterm premature rupture of the membranes.

The study objectives here were to characterize the vaginal microbiota of women presenting with PPROM and throughout latency, and to determine if vaginal microbial profile at the time of membrane rupture predicts latency duration and/or perinatal outcomes. Results of the study show that women with PPROM had mixed, highly variable vaginal microbiota but the specific type of microbiome profile at PPROM did not correlate with latency duration. Additional associations between the presence of Mollicutes, lower gestational age at delivery, and lower birth weight were detected. The presence of two taxa associated with vaginal dysbiosis, *Megasphaera* sp. type I and *Prevotella* spp., in all samples warrants further investigation.

## Materials and Methods

### Study design

The study received ethics approval from the University of British Columbia Children’s and Women’s Research Ethics Board (certificate no. H08-01904).

This was designed as a prospective exploratory study of women with PPROM between 24^+0^ and 33^+6^ weeks gestational age (GA), recruited at BC Women's Hospital, Vancouver, Canada. Women were approached on presentation with suspected PPROM. Verbal consent at the time of speculum examination was approved allowing confirmation of rupture of membranes and permitting immediate sample collection. Subsequently, women were invited to participate through fully informed written consent; if they declined, samples were discarded. Inclusion criteria were: adequate comprehension of English to give written informed consent, singleton gestation, no known fetal anomalies or complications distinct from PPROM, and no maternal or neonatal indication for iatrogenic PPROM. Ruptured membranes were confirmed by evidence of vaginal fluid pooling, a positive nitrazine test (elevated pH), and a positive ferning test [22].

Three swabs were collected from each woman at enrolment: a cervical swab for *Chlamydia trachomatis* and *Neisseria gonorrhea* NAT [23, 24], and two vaginal swabs from the posterior fornix and lateral vaginal wall, one for Gram stain assessment using Nugent score [25], and one for microbiome characterization. Swabs for microbiome analysis were stored, dry, at -80°C until processing. At weekly intervals, women were reviewed, clinical status recorded and additional vaginal samples obtained. This continued until delivery. Vaginal swabs for Gram stain and microbiome analysis were obtained prior to delivery where feasible.

Demographic and clinical characteristics were collected from patient charts. Clinical chorioamnionitis was diagnosed based on standard criteria of maternal pyrexia of >38°C and at least two of the following: fetal tachycardia >160 bpm, maternal tachycardia >120 bpm, leukocyte count >14000 cells, uterine tenderness and/or foul amniotic fluid. Histological chorioamnionitis was diagnosed based on placental pathology.

Neonatal outcome data collected included APGAR score [26], birth weight, gender, admission to the Neonatal Intensive Care Unit (NICU) and morbidity assessment index for newborns (MAIN) score [27].

### Microbiome profiling

DNA was extracted from vaginal swabs using a magnetic bead-based kit (MagMAX, Life Technologies, Burlington, ON). PCR amplicon libraries were created as described previously using primers targeting the *cpn*60 universal target [28]. Sequencing was performed on the 454 GS FLX Titanium and GS Junior sequencing platforms.

High quality reads were processed as previously described using the microbial Profiling Using Metagenomic Assembly (mPUMA) pipeline [29]. Taxonomic identification of operational taxonomic units (OTU) was accomplished through comparison of OTU sequences to the cpnDB_nr reference database [30] (downloaded from www.cpndb.ca). For most analyses, OTU sequences with the same nearest reference database sequence were pooled as nearest neighbour "species" and their abundances combined accordingly.

### Detection of Mollicutes

Mollicutes (*Mycoplasma* and *Ureaplasma*) were detected by genus-specific, conventional semi-nested PCR targeting the 16S rRNA gene [31]. The primary PCR targeted a 700 bp portion of the 16S rRNA gene using primers GPO-1 and MGSO [31]. PCR was performed under the following conditions: 40 cycles of 94°C for 30 s, 64°C for 30 s, and 72°C for 60 s. The secondary PCR used primers My-ins [32] and MGSO, and 2 μl of the primary PCR product as template. Thermocycling parameters included 35 cycles of 94°C for 30 s, 60°C for 30 s, and 72°C for 60 s.

*Ureaplasma* species (*U*. *parvum* and *U*. *urealyticum*) were detected using a conventional PCR based on the multiple-banded antigen gene with primers UMS-125 and UMA226, which yield products of two different sizes depending on the target species: 403 bp (*Ureaplasma parvum*) or 443 bp (*Ureaplasma urealyticum*) [33, 34]. Specific detection of *Mycoplasma genitalium* was done using a species-specific conventional PCR targeting the 16S rRNA gene [35]. *M*. *genitalium* genomic DNA for use as a positive control was obtained from the American Type Culture Collection (ATCC, Manassas, VA).

Mollicutes-specific 16S rRNA gene PCR amplicons from ten samples obtained from eight women were conveniently chosen and pooled and pyrosequenced as described for *cpn*60 amplicon libraries in order to gain species level identification of taxa detected by this family-specific PCR.

### Statistical analysis

Demographic and clinical variables of the study cohort were investigated using non-parametric, descriptive statistics (IBM SPSS, version 21).

#### Microbiome data

Shannon’s diversity index, Chao1 estimated number of species and jackknifed Bray-Curtis dissimilarity were calculated using the Quantitative Insights Into Microbial Ecology (QIIME) package [36]. These indexes were bootstrapped 100 times at 1000 reads per sample or their sample maximum when <1000 reads were available. Rarefaction plots of these alpha diversity measures were generated to ensure an adequate sampling depth for each sample had been achieved. Average linkage hierarchical clustering was performed based on the proportion of each nearest neighbour species per sample using the vegan package in R [37].

## Results

### Maternal clinical data

Women presenting with PPROM between September 2010 and December 2012, who were >18 years of age and able to consent, were approached for participation in the study. Women with multiple gestations, stillbirths, known fetal anomalies or indications for immediate delivery were excluded. At time of study closure 51 women consented and agreed to provide samples. Of these, 17% had assessment vaginal samples that had insufficient cells present to assess by standard microscopy and 15 did not have adequate DNA extracted for evaluation. This resulted in 36 women for whom clinical and microbiologic data was available. Demographic and clinical characteristics of the women are provided in Table 1. Clinical chorioamnionitis was diagnosed in 35.5% (11/36) of women. Histopathological chorioamnionitis was detected in 67.7% (21/36) of women. There was no difference in clinical characteristics between those women for whom there was adequate microbiologic data and those excluded (data not shown).

**Table 1:** Demographic and clinical data for study participants (n = 36). BMI; body mass index, BV; bacterial vaginosis, STI; sexually transmitted infection, Continuous variables are reported as means ± standard deviation [range], Counts are reported as median [IQR], Categorical variables are reported as N [%].

Age	32.92 ± 4.83 [22–40]
Pre-pregnancy BMI	23.69 ± 4.56 [17.6–37.3]
Gestational diabetes	5 [13.9%]
**Ethnicity**	
Asian	9 [25%]
South Asian	1 [2.8%]
White	20 [55.6%]
Aboriginal	3 [8.3%]
Other	3 [8.3%]
**Substance use during pregnancy**	
Smoking	7 [19.4%]
Alcohol use	4 [11.1%]
**Sexual history**	
Marital Status	
Partnered	32 [88.9%]
Single	4 [11.1%]
One sexual partner during pregnancy (n = 31)	31 [100%]
Vaginal intercourse during pregnancy (n = 34)	31 [91.2%]
Oral sex received during pregnancy (n = 34)	11 [32.4%]
**Pregnancy history**	
Previous pregnancy	36 [100%]
Number of previous pregnancies (Gravida)	2 [1.8–3.3]
Number of live births	1 [0–1.3]
Any previous preterm birth	5 [13.9%]
**BV and STI history during pregnancy**	
Diagnosed with BV (n = 34)	2 [5.9%]
Diagnosed with yeast	7 [19.4%]
Diagnosed with UTI	6 [16.7%]
Diagnosed with Group B *Streptococcus* (n = 34)	2 [5.9%]
Diagnosed with *Chlamydia* (n = 34)	0
Diagnosed with *Gonorrhea* (n = 34)	0
**Antimicrobial use for genital tract infection during pregnancy (n = 31)**	5 [13.9%]
**Other**	
Feminine wipes or deodorant products (n = 34)	5 [14.7%]

**Fig 1:**
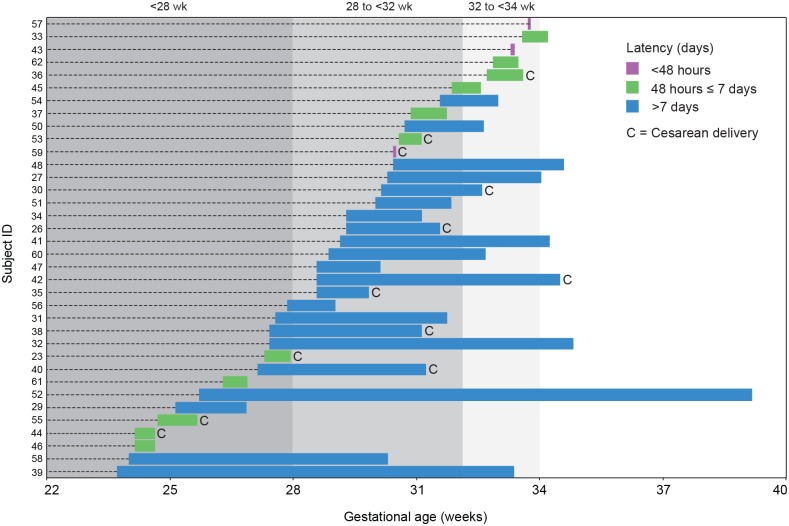
GA at PPROM, latency duration and delivery mode for 36 women enrolled in the study. GA ranges corresponding to extremely (<28 weeks), very (28 to <32 weeks) or moderately (32 to <34 weeks) preterm are indicated by grey shading.

Mean GA at PPROM was 29 weeks; mean GA at delivery was 32 weeks; mean latency period was 18 days (Fig 1, Table 2). As expected, latency (days) was negatively correlated (ρs = -0.390, n = 36, p = 0.019) with GA at PPROM because the number of potential latency days decreases with increasing GA at PPROM. Since latency duration was a variable of particular interest in this study, we considered both absolute latency duration (< 48 hours, between 48 hours and ≤ 7 days, and > 7 days following membrane rupture), and latency duration as a proportion of maximum possible latency. According to the guidelines of the American College of Obstetrics and Gynecologists [38] and Society of Obstetrics and Gynecologists of Canada [5], conservative/expectant management of PPROM is recommended for gestational ages 24 to 33 weeks and induction of labour is warranted after 34 weeks. Thus, the composite variable, "proportional latency", was calculated using the following equation:

$$\text{Proportional latency =}\frac{{\text{GA}}_{\text{delivery}}{\text{ - GA}}_{\text{PPROM}}}{{\text{34 - GA}}_{\text{PPROM}}}$$

**Table 2:** Summary of PPROM to delivery timelines.

	No. of women (/36)
**Gestational age at PPROM**	
Extremely preterm (<28 weeks)	14
Very preterm (28 to <32 weeks)	17
Moderate preterm (32 to <34 weeks)	5
**Absolute latency**	
<48 hours	3
Between 48 hours and < 7days	11
> 7 days	22
**Proportional latency**	
0–0.33	15
0.34–0.66	10
0.67–1.0	11
**Mode of delivery**	
Vaginal delivery	24
Cesarean delivery	12

Clinical variables were analyzed for relationship to latency and we found that proportional and absolute latency were negatively correlated with body mass index (BMI) (ρs = -0.511, n = 31, p = 0.003).

### Samples for microbiome analysis

Over the study duration, 82 vaginal samples were collected. Vaginal samples collected within 24 hours following PPROM and prior to administration of antibiotics were categorized as T_0_ samples (time zero, n = 24). Subsequently, weekly samples were collected until delivery, when a delivery sample was collected if possible. Average number of samples per woman was two (range 1–7).

### Gram stain results

Nugent’s scoring of 19/24 T_0_ samples were predominantly inconsistent with bacterial vaginosis (BV) (≥ 7), with 73.9% (17/23) having normal scores (<4), and 8.7% (2/23) having intermediate scores (4–6). One sample had missing data. Altogether, 43 weekly vaginal Gram stains were evaluated. Only one was consistent with BV, 13 (30.2%) were normal and 25 (58.1%) had intermediate scores. Of the seven delivery samples, five had intermediate and two had normal scores.

### Microbial profiles

For the 70 vaginal swab samples which yielded sufficient PCR product for *cpn*60-based microbiome profiling, an average of 12,675 *cpn*60 sequence reads were obtained per sample (range 36–99,358; median 6,296). One sample (week 2, woman 60) yielded only 36 sequence reads and was excluded from statistical analyses. Rarefaction analysis of alpha diversity measures (Shannon’s diversity index and Chao1 estimated number of species) indicated that all other samples had received adequate coverage for inclusion in further analysis. Raw sequence data files for the samples described in this study were deposited to the NCBI Sequence Read Archive (Accession SRP077099, BioProject PRJNA326844).

**Fig 2:**
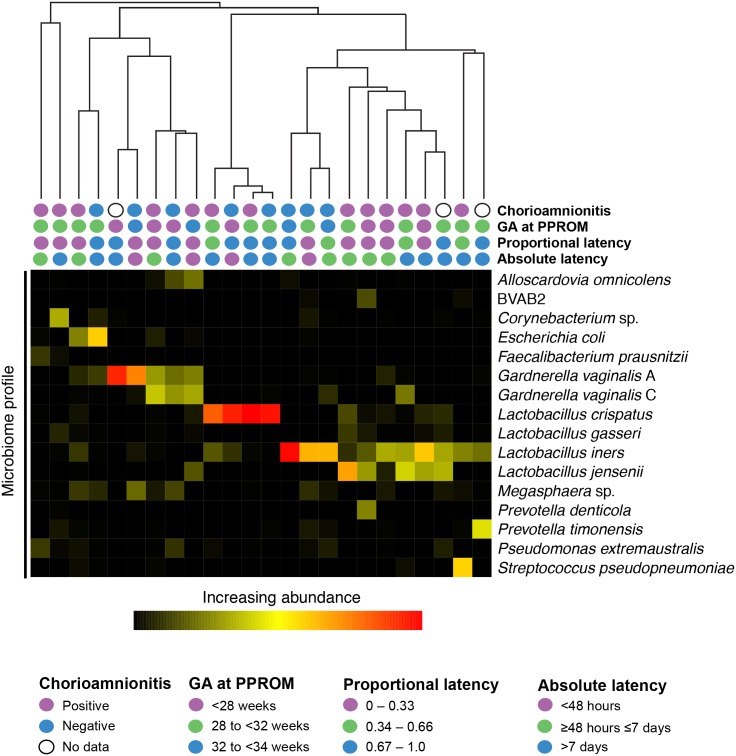
Average linkage hierarchical clustering of *cpn*60 based microbiome profiles of T_0_ vaginal samples from 24 women. Nearest neighbour species representing at least 10% of the microbiome of at least one woman are included.

T_0_ microbiome profiles clustered into profile types, each dominated by one or two species including *Lactobacillus crispatus*, *L*. *iners*, *P*. *timonensis*, *Gardnerella vaginalis* (subgroups A and C), *Corynebacterium* sp. and *Escherichia coli* (Fig 2). Half (12/24) of T_0_ samples were dominated (≥50% of the microbiome) by one or more species of *Lactobacillus*. In eight cases, a single *Lactobacillus* sp. comprised at least 50% of the microbiome: *L*. *crispatus* (n = 4), *L*. *iners* (n = 3) and *L*. *jensenii* (n = 1), corresponding to previously described Community State Types (CST) I, III and V, respectively [39, 40]. Profiles dominated by *G*. *vaginalis* subgroup A (n = 2) correspond to CST IVC [39]. The remaining profiles included mixtures of *G*. *vaginalis* A and C or were dominated by other non-*Lactobacillus* species and more closely resembled CST IVA, which is defined by its heterogeneity [39].

Shannon’s diversity index and Chao1 diversity of T_0_ samples were not significantly different between proportional latency and absolute latency groups (Table 2) (Kruskal-Wallis, p > 0.05). Microbiome profile clusters were not statistically associated with outcomes of GA at PPROM, latency (absolute or proportional), or chorioamnionitis (clinical or histopathological) (Fig 2).

### Core microbiome and highly prevalent organisms

A total of 670 OTUs were detected in 70 vaginal samples with 9 OTUs accounting for >50% of sequence reads obtained. Twenty nearest neighbour taxa accounted for 77% of the data (S1 Table). The most abundant OTU (12.4% of the sequence reads) was 97.1% identical to *Prevotella timonensis* (S1 Table) and was detected in 34/36 women (64/70 samples). An OTU sequence with 99.6% identity to *Megasphaera* sp. UPII 199–6, a type I *Megasphaera*, was detected in all 70 vaginal samples (comprising 0.0076% to 22.6% of individual microbiome profiles). OTU sequences with best matches to any *Megasphaera* spp. were combined with reference sequences to construct the phylogenetic tree (S1 Fig). Four additional OTU sequences clustered with *M*. *micronuciformis*, *M*. *elsdenii*, and *Megasphaera* sp. BV3C16-1.

OTU sequences identical to *L*. *iners* were detected in all T_0_ samples (n = 24). In three samples, *L*. *iners* was dominant, accounting for ≥50% of total reads.

*Prevotella* spp. were detected in all vaginal samples, and were dominant (accounting for ≥50% of reads) in 1/24 T_0_ samples and 10/46 weekly samples. Fourteen different *Prevotella* spp. were detected in all T_0_ samples, along with 15 other *Prevotella*-like OTU sequences with weaker (80–95%) similarity to reference sequences, suggesting possibly novel taxa (S2 Fig). The most prevalent species were *P*. *timonensis* and *P*. *bivia*, detected in 92% and 42% of T_0_ samples respectively.

### Mollicutes

**Fig 3:**
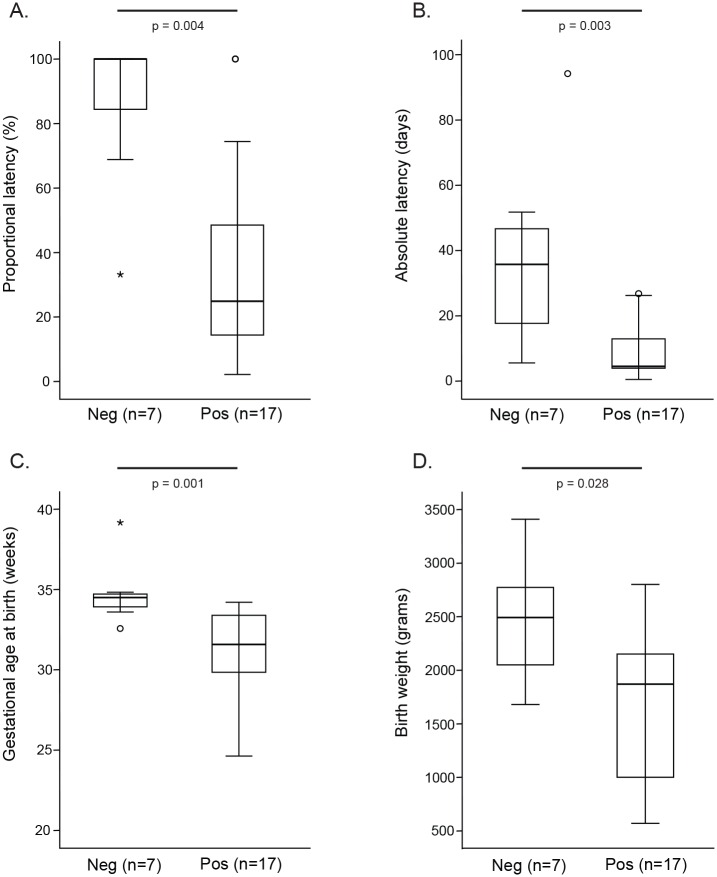
Proportional latency (A), absolute latency (B), GA at birth (C) and infant birthweight (D) for women with Mollicutes PCR positive or negative T_0_ vaginal samples. Distributions were compared using a Mann-Whitney U test with *P* <0.05 considered significant.

Mollicutes (*Mycoplasma* and/or *Ureaplasma*) were detected by family-specific PCR in 81% (29/36) of women, *Ureaplasma* spp. in 53% (19/36), *U*. *parvum* in 39% (14/36) and *U*. *urealyticum* in 17% (5/36) of women. Both *Ureaplasma* species were detected together in only one sample. When only T_0_ samples were considered, 70.8% (17/24) were PCR positive for Mollicutes. Both proportional and absolute latency were significantly shorter in women whose T_0_ samples were PCR positive for Mollicutes compared to women who were Mollicutes negative (Fig 3, panels A, B). Women whose T_0_ samples were positive for Mollicutes had significantly lower GA at delivery (Fig 3, panel C), delivering correspondingly lower birth weight infants (Fig 3, panel D). In order to gain species-level information about *Mycoplasma* and to confirm the specificity of the PCR, we performed pyrosequencing of Mollicutes-specific 16S rRNA secondary PCR products pooled from ten samples (representing eight women). All reads (n = 28,278) could be classified as *M*. *hominis*, *U*. *parvum* or *U*. *urealyticum*. No reads were classified as *M*. *genitalium*. To rule out the possibility that the Mollicutes specific PCR failed to amplify *M*. *genitalium*, we confirmed its performance using purified genomic DNA from *M*. *genitalium* (ATCC, Manassas, VA). For additional confirmation of the absence of *M*. *genitalium*, we subsequently screened all samples with an *M*. *genitalium* species-specific PCR as described in the Methods. All samples were PCR negative for *M*. *genitalium*.

### Instability of the vaginal microbiota over the latency period

**Fig 4:**
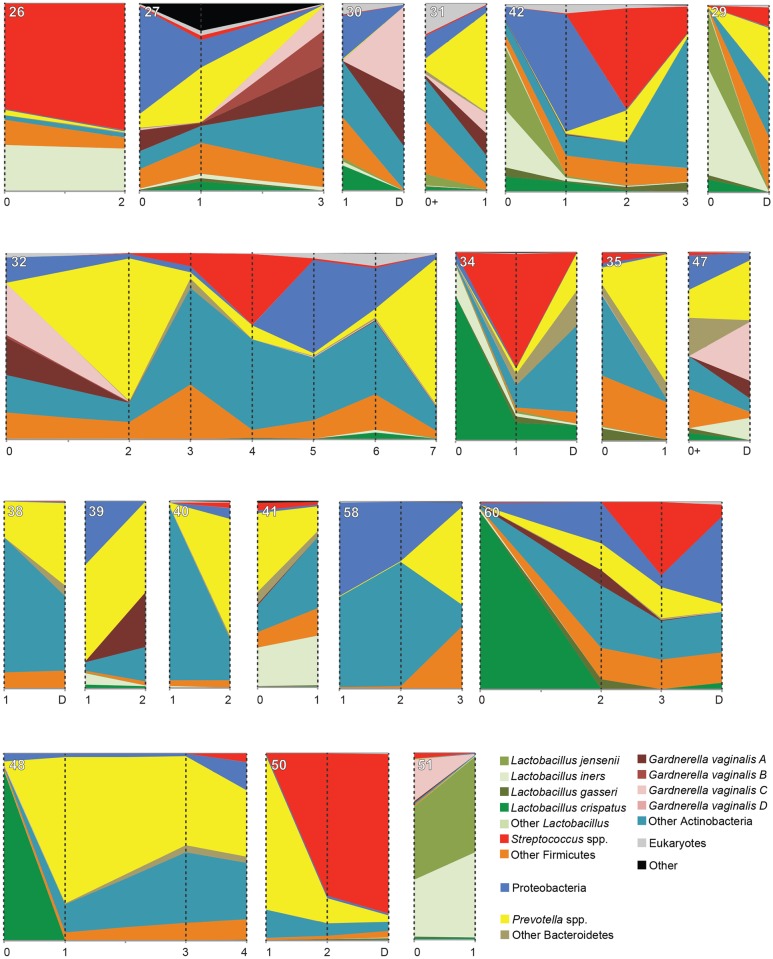
Vaginal microbiome profiles over the post-PPROM latency period. Data is presented as proportion of the total sequence reads obtained for each sample, with the height of the ordinate corresponding to 100%. Sampling times are indicated with vertical broken lines, and collection time (weeks) for each sample is indicated on the abscissa. Samples indicated as collected at "0+" were collected within 3 days following PPROM, but were post-antibiotic treatment. Sample identification numbers appear in the upper left corner of each panel. The legend includes nearest neighbour species that account for at least 10% of the sequence reads in at least one sample. All women received one or more broad-spectrum antibiotics including Ampicillin, Erythromycin, Amoxicillin, Metronidazole, Clindamycin, Cefazolin, Nitrofurantoin, and/or Penicillin G.

All women received one or more broad-spectrum antibiotics (range 1–7 types). The variations in the types and duration of antibody therapy in this small cohort size did not allow for any associations to be drawn between antibiotic administration and microbiome changes in the latency period. In all 19 women for whom multiple samples were available, the apparent composition of the vaginal microbiota changed dramatically over the latency period (Fig 4). In 4/5 cases where *Lactobacillus* dominated the T_0_ microbiome, its proportional abundance had decreased substantially by the subsequent sample time point. Eight of 19 women for whom multiple samples were available had one or more samples dominated (≥50% of the microbiome) by *Prevotella* spp. (Fig 4). Of the remaining 11 women, five had *Prevotella* spp. as the proportionally most abundant taxon in at least one sample.

When the PPROM cohort was compared to a cohort of healthy, non-pregnant women for whom longitudinal data was available [41], the Bray-Curtis dissimilarity between samples within PPROM cohort individuals was significantly greater (ANOVA, *F*_1, 112_ = 95.78, p < 0.0001) than the average dissimilarity for samples within individuals in the healthy, non-pregnant cohort, indicating greater instability of PPROM associated microbiomes (S3 Fig).

### Neonatal outcomes

Neonatal outcomes are summarized in Table 3. APGAR score at 1 minute was positively correlated to gestational age at delivery (*ρs* = 0.631, n = 35, p = 0.0001), as was APGAR score at 5 minutes (*ρs* = 0.592, n = 35, p = 0.0001).

**Table 3:** Neonatal outcomes. Continuous variables are reported as means ± 95% CI [range]. Categorical variables are reported as N [%].

**Gender**	
Male	25
Female	11
**Birth weight (grams)**	1812.9 [570–3410]
Extremely low birth weight (<1000 g)	6 [16.7%]
Very low birth weight (<1500 g)	4 [11.1%]
Low birth weight (<2500 g)	20 [55.6%]
Normal	6 [16.7%]
**Small for gestational age** (n = 35)	
Moderate (<10^th^ percentile)	2 [5.7%]
Severe (<3^rd^ percentile)	2 [5.7%]
**Bronchopulmonary dysplasia** (n = 32)	
None	23 [71.8%]
Mild	4 [12.5%]
Severe	3 [9.4%]
**Apgar 1 minute score** (n = 35)	
<7 (Critical and low)	19
7–10 (Normal)	16
**Apgar 5 minute score** (n = 35)	
<7 (Critical and low)	5
7–10 (Normal)	30
**MAIN score**	563.2 [0–1878]
**NICU admission (n = 34)**	23 [68]
Days of stay in NICU	24.57 [0.81–137.74]

All but one infant born to women in this study survived. One infant, delivered vaginally at 25 weeks after a three-day latency period (birth weight 570 g), died of late onset sepsis at 46 days of life. Blood culture of the infant tested positive for *E*. *coli* at 12 days of life. *E*. *coli* was present in the vaginal microbiome of the mother (T_0_ vaginal sample, detected at 0.02% of the total microbiome). The mother’s microbiome was dominated by *L*. *iners* (32% of reads), followed by *G*. *vaginalis*. The vaginal T_0_ sample was positive for Mollicutes and *U*. *urealyticum*.

## Discussion

### Main findings

Highly diverse vaginal microbiomes were found in women at time of rupture of membranes with variability throughout latency. Of note, none of the microbiomes represented classic bacterial vaginosis, nor was a specific microbiome profile type associated with latency or neonatal outcomes. However, it was notable that two taxa associated with vaginal dysbiosis, *Megasphaera* sp. type I, and *Prevotella* spp. were found in all samples. Presence of *Mycoplasma* and/or *Ureaplasma* was associated with lower gestational age and delivery and lower birth weight.

### Strengths and limitations

The strength of this study is that it is an in-depth prospective study which characterizes the microbiome following preterm premature rupture of membranes, providing new insights into the microbial profile of this fastidious, hard to culture bacterial community in these high risk pregnancies. The main limitations of this study are the relatively small number of women followed and the lack of pre-rupture samples as prediction of PPROM is very difficult. The standard use of broad-spectrum antibiotics in the context of PPROM means that the natural changes of the microbiome during latency can no longer be evaluated. Ongoing studies by our group are assessing the microbiomes of women in pregnancy, to establish the pre-rupture microbiome and to try to ascertain causality rather than association of microbial profiles with adverse perinatal outcomes.

### Interpretation

Understanding the microbiome associated with PTB and PPROM is critical to creating strategies to prevent this reproductive outcome and to determine when to initiate delivery. To date, characteristics of the vaginal microbiome during the latency period following PPROM have been poorly understood. Here, the diversity and instability of women’s microbiomes following PPROM was striking. Hierarchical clustering of T_0_ profiles did not reveal specific associations between microbiome profiles and gestational age at PPROM, latency duration, or development of chorioamnionitis. However, although the full profile did not predict outcomes, presence of Mollicutes was a strong predictor of shorter latency. Additionally, analysis of weekly samples collected during the latency period revealed an unstable microbiome different from classic bacterial vaginosis but consistent with a dysbiosis. Of note, despite antibiotic therapy being given to prolong pregnancy, there was not a restoration of a normal *Lactobacillus* dominant microbiome in these women during the latency period. In fact, in 4/5 cases where *Lactobacillus* dominated the microbiome at PPROM, its proportional abundance decreased during subsequent latency time points. In the original studies of the use of antibiotics for latency, there were no in depth genomic based analyses available of the microbiome pre and post antibiotic therapy.

Historically, BV has been identified as a risk factor for PTB and PPROM [8, 42–46]. Here, none of the T_0_ samples had Nugent scores consistent with BV. Amniotic fluid leaking after membrane rupture may have resulted in a more dilute sample, reducing bacteria available for scoring. However, a lack of consistency of T_0_ microbiota with BV was also apparent in the sequence-based profiles, with few T_0_ sample microbiome profiles dominated by *G*. *vaginalis* (5/24) or *Atopobium vaginae* (0/24), which are strongly associated with a Nugent score diagnosis of BV [47]. Classic BV profiles were thus not the dominant profile at onset or shortly after PPROM.

Approximately half of the T_0_ sample profiles were dominated by *Lactobacillus*. Among these, the most common *Lactobacillus* was *L*. *iners*, either alone or in combination with *L*. *jensenii*. *L*. *iners* is associated both with normal and BV microbiota, and is reported as a dominant organism when the vaginal microbiota is in a transition state, either from normal to BV or vice versa [48–50], while *L*. *iners*-dominated microbiomes have been associated with spontaneous PTB [51]. The hemolytic and mucinolytic potential of *L*. *iners* could be involved in its survival during disturbed vaginal conditions, and may be suggestive of its pathogenic potential [52]. In contrast, longitudinal stability of vaginal microbiota in pregnant women with term deliveries has been associated with *L*. *crispatus* dominated profiles [19].

*Prevotella* spp. was detected in all samples from all women and dominated some profiles either at T_0_ or subsequent time points during latency. In contrast, a *cpn*60-based study of 91 vaginal samples from clinically healthy, non-pregnant, Canadian women detected *Prevotella* in only 57% of samples, with the most frequently detected species (*P*. *timonensis*) present in 45% of samples [41]. Here, we identified 14 known species based on comparison of OTU sequences to the cpnDB reference database [HILL et al. 2004], which currently contains *cpn*60 sequences for 46 *Prevotella* spp. An additional 15 *Prevotella*-like OTU sequences with no significant similarity to these 46 species, may include novel taxa. Given the suggested role of *Prevotella* in synergistic relationships with BV associated organisms [53, 54] and their production of lipopolysaccharide in the vaginal environment [55], the prevalence and diversity of *Prevotella* in PPROM associated vaginal microbiota warrants further investigation.

OTU sequences corresponding to *Megasphaera* type 1 were detected in all samples. *Megasphaera* spp. have been associated with BV [56, 57]. Two OTU sequences clustered with *M*. *micronuciformis* F0359, an obligate anaerobe isolated from the human oral cavity in the Human Microbiome Project. Identification of bacterial species associated with the oral microbiome in amniotic fluid of women who experience PTB has been observed previously, although the significance of this relationship and whether it is indicative of transfer of organisms is unknown [58, 59].

Genital *Mycoplasma* and *Ureaplasma* have been previously associated with adverse maternal/fetal outcomes [60–62]. Cervical colonization of genital mycoplasmas is associated with PTB and PPROM. These organisms have been isolated from amniotic fluid and the chorioamnion of women with PPROM and preterm labour [15, 63]. Since *cpn*60 genes are known to be absent in some Mollicutes [64], specific PCR was used to detect these organisms. Here, 80% of women were PCR positive for *Mycoplasma* and/or *Ureaplasma*, at the higher end of the range reported in pregnant women (10–84%) [65–68]. *M*. *genitalium*, an organism associated with cervicitis and infertility issues in women [68–70], was not detected in any woman in this study. This population had low rates of cervicitis, comparable to other studies that either did not detect the species in amniotic fluid or were not able to demonstrate associations with adverse pregnancy outcomes [68, 71]. Here, the presence of vaginal Mollicutes was associated with significantly lower GA at delivery, and correspondingly lower birth weight, supporting previous suggestions of a potential role for these organisms in adverse pregnancy outcomes.

Antibiotic and steroid administration, given as part of standard guidelines for PPROM <32 weeks [5], may have contributed to the diverse and abnormal microbiome profiles observed through latency. Another potential contributor is leakage of amniotic fluid (pH 7 to 7.5) [72] through the normally acidic vaginal environment. These factors likely contributed to the rapid transition to an abnormal microbial profile during latency in the four women who had *Lactobacillus* dominated profiles at PPROM.

Perhaps most important to note was the microbiota instability over the latency period. The microbial profiles and quantitative assessment of ecological distance between samples within and between women are in stark contrast to the generally stable microbial community observed in longitudinal studies of healthy, non-pregnant [40, 41] and pregnant women [73]. Stability in terms of maintenance of a core set of metabolic capabilities of the human microbiome is correlated to health, even when the community structure varies in its taxonomic composition [74]. It is highly likely that the antibiotic and steroid therapy, used in a standard fashion, may be playing a substantive role, but is affecting women in different ways.

Taken together, results here provide an unprecedented characterization of the vaginal microbiota following PPROM, and support previous suggestions of a role for Mollicutes in adverse outcomes associated with PPROM. Results also demonstrate the value of application of culture-independent methods for microbial profiling to better understand this important clinical problem.

## Conclusions

Women with PPROM had mixed, highly variable vaginal microbiota but the specific type of microbiome profile at PPROM did not correlate with latency duration. The highly unstable vaginal microbiota of women in this study demonstrates the need for more intense study of the relationship of genital tract microbiota with PPROM, including functional analysis of these microbial communities. Future work should involve larger studies including sampling before/after membrane rupture, to ascertain the predisposing microbiome leading to membrane rupture.

## Supporting Information

S1 Fig Phylogenetic tree of *Megasphaera*-like study sequences (represented by circles, n = 6) combined with *Megasphaera* reference sequences. The tree is based on a 315 bp alignment and was constructed using the F84 distance algorithm followed by neighbour joining using the PHYLIP software package (Felsenstein J. PHYLIP—phylogeny inference package (version 3.2). Cladistics. 1989;5: 164–6). Bootstrap values (>50%) are indicated at nodes.
(TIF)

S2 Fig Phylogenetic tree of *Prevotella*-like study sequences and *Prevotella* reference sequences. Study sequences detected in T_0_ samples are indicated by triangles. Sequences represented by circles were detected only in weekly or delivery vaginal samples. The tree is based on a 300 bp alignment and was constructed using the F84 distance algorithm followed by neighbour joining using the PHYLIP software package (Felsenstein J. PHYLIP—phylogeny inference package (version 3.2). Cladistics. 1989;5: 164–6). Only 28 of the 36 identified *Prevotella-*like OTU could be included in the tree since the remaining sequences did not provide sufficient overlap to be included in the alignment. Bootstrap values (>50%) are indicated at node.
(TIF)

S3 Fig Bray-Curtis distances between vaginal microbiome profiles of women with PPROM, and clinically healthy, non-pregnant reproductive aged women. (TIF)

S1 Table Prevalence of nearest neighbour species accounting for at least 1% of the total sequence reads in the study. (DOC)

## References

[1] Blencowe H, Cousens S, Chou D, Oestergaard M, Say L, Moller AB, et al Born too soon: the global epidemiology of 15 million preterm births. Reprod Health. 2013;10 Suppl 1(S2. 24625129 10.1186/1742-4755-10-S1-S2 PMC3828585

[2] Goldenberg RL, Culhane JF, Iams JD, Romero R. Epidemiology and causes of preterm birth. Lancet. 2008;371(9606): 75–84. 10.1016/S0140-6736(08)60074-4 18177778 PMC7134569

[3] Mercer BM. Preterm premature rupture of the membranes. Obstet Gynecol. 2003;101(1): 178–93. 12517665 10.1016/s0029-7844(02)02366-9

[4] Frenette P, Dodds L, Armson BA, Jangaard K. Preterm prelabour rupture of membranes: effect of latency on neonatal and maternal outcomes. J Obstet Gynaecol Can. 2013;35(8): 710–7. 24007706 10.1016/S1701-2163(15)30861-6

[5] Yudin MH, van Schalkwyk J, Van Eyk N, Boucher M, Castillo E, Cormier B, et al Antibiotic therapy in preterm premature rupture of the membranes. J Obstet Gynaecol Can. 2009;31(9): 863–7, 8–74. 19941711 10.1016/S1701-2163(16)34305-5

[6] Simhan HN, Canavan TP. Preterm premature rupture of membranes: diagnosis, evaluation and management strategies. BJOG. 2005;112 Suppl 1(32–7. 15715592 10.1111/j.1471-0528.2005.00582.x

[7] Dare MR, Middleton P, Crowther CA, Flenady VJ, Varatharaju B. Planned early birth versus expectant management (waiting) for prelabour rupture of membranes at term (37 weeks or more). Cochrane Database Syst Rev. 2006;1): CD005302. 10.1002/14651858.CD005302.pub2 16437525

[8] Hillier SL, Nugent RP, Eschenbach DA, Krohn MA, Gibbs RS, Martin DH, et al Association between bacterial vaginosis and preterm delivery of a low-birth-weight infant. The Vaginal Infections and Prematurity Study Group. N Engl J Med. 1995;333(26): 1737–42. 10.1056/NEJM199512283332604 7491137

[9] Leitich H, Bodner-Adler B, Brunbauer M, Kaider A, Egarter C, Husslein P. Bacterial vaginosis as a risk factor for preterm delivery: a meta-analysis. Am J Obstet Gynecol. 2003;189(1): 139–47. 12861153 10.1067/mob.2003.339

[10] Parry S, Strauss JF 3rd. Premature rupture of the fetal membranes. N Engl J Med. 1998;338(10): 663–70. 10.1056/NEJM199803053381006 9486996

[11] Locksmith G, Duff P. Infection, antibiotics, and preterm delivery. Semin Perinatol. 2001;25(5): 295–309. 11707017 10.1053/sper.2001.27163

[12] Goldenberg RL, Culhane JF. Infection as a cause of preterm birth. Clin Perinatol. 2003;30(4): 677–700. 14714919 10.1016/s0095-5108(03)00110-6

[13] Goldenberg RL, Culhane JF, Johnson DC. Maternal infection and adverse fetal and neonatal outcomes. Clin Perinatol. 2005;32(3): 523–59. 10.1016/j.clp.2005.04.006 16085019 PMC7119141

[14] Read JS, Klebanoff MA. Sexual intercourse during pregnancy and preterm delivery: effects of vaginal microorganisms. The Vaginal Infections and Prematurity Study Group. Am J Obstet Gynecol. 1993;168(2): 514–9. 8438920 10.1016/0002-9378(93)90484-z

[15] Wang X, Buhimschi CS, Temoin S, Bhandari V, Han YW, Buhimschi IA. Comparative microbial analysis of paired amniotic fluid and cord blood from pregnancies complicated by preterm birth and early-onset neonatal sepsis. PLoS One. 2013;8(2): e56131 10.1371/journal.pone.0056131 23437088 PMC3577789

[16] Fortner KB, Grotegut CA, Ransom CE, Bentley RC, Feng L, Lan L, et al Bacteria localization and chorion thinning among preterm premature rupture of membranes. PLoS One. 2014;9(1): e83338 10.1371/journal.pone.0083338 24421883 PMC3885429

[17] DiGiulio DB, Romero R, Kusanovic JP, Gomez R, Kim CJ, Seok KS, et al Prevalence and diversity of microbes in the amniotic fluid, the fetal inflammatory response, and pregnancy outcome in women with preterm pre-labor rupture of membranes. Am J Reprod Immunol. 2010;64(1): 38–57. 10.1111/j.1600-0897.2010.00830.x 20331587 PMC2907911

[18] Jones HE, Harris KA, Azizia M, Bank L, Carpenter B, Hartley JC, et al Differing prevalence and diversity of bacterial species in fetal membranes from very preterm and term labor. PLoS One. 2009;4(12): e8205 10.1371/journal.pone.0008205 19997613 PMC2785424

[19] Romero R, Hassan SS, Gajer P, Tarca AL, Fadrosh DW, Bieda J, et al The vaginal microbiota of pregnant women who subsequently have spontaneous preterm labor and delivery and those with a normal delivery at term. Microbiome. 2014;2(18 10.1186/2049-2618-2-18 24987521 PMC4066267

[20] Nelson DB, Hanlon A, Nachamkin I, Haggerty C, Mastrogiannis DS, Liu C, et al Early pregnancy changes in bacterial vaginosis-associated bacteria and preterm delivery. Paediatr Perinat Epidemiol. 2014;28(2): 88–96. 10.1111/ppe.12106 24405280 PMC4031320

[21] Wen A, Srinivasan U, Goldberg D, Owen J, Marrs CF, Misra D, et al Selected vaginal bacteria and risk of preterm birth: an ecological perspective. J Infect Dis. 2014;209(7): 1087–94. 10.1093/infdis/jit632 24273044 PMC3952673

[22] Cunningham FG, Levono KJ, Bloom SL, Hauth JC, Rouse DJ, Spong CY. Williams Obstetrics. 23rd Edition New York: McGraw-Hill Medical; 2010.

[23] Van Dyck E, Ieven M, Pattyn S, Van Damme L, Laga M. Detection of *Chlamydia trachomatis* and *Neisseria gonorrhoeae* by enzyme immunoassay, culture, and three nucleic acid amplification tests. J Clin Microbiol. 2001;39(5): 1751–6. 10.1128/JCM.39.5.1751-1756.2001 11325985 PMC88020

[24] Cook RL, Hutchison SL, Ostergaard L, Braithwaite RS, Ness RB. Systematic review: noninvasive testing for *Chlamydia trachomatis* and *Neisseria gonorrhoeae*. Ann Intern Med. 2005;142(11): 914–25. 15941699 10.7326/0003-4819-142-11-200506070-00010

[25] Nugent RP, Krohn MA, Hillier SL. Reliability of diagnosing bacterial vaginosis is improved by a standardized method of gram stain interpretation. J Clin Microbiol. 1991;29(2): 297–301. 1706728 10.1128/jcm.29.2.297-301.1991 PMC269757

[26] Queiros da Mota V, Prodhom G, Yan P, Hohlfheld P, Greub G, Rouleau C. Correlation between placental bacterial culture results and histological chorioamnionitis: a prospective study on 376 placentas. J Clin Pathol. 2013;66(3): 243–8. 10.1136/jclinpath-2012-201124 23268318

[27] American Academy of P, Committee on F, Newborn, American College of O, Gynecologists, Committee on Obstetric P. The Apgar score. Adv Neonatal Care. 2006;6(4): 220–3. 10.1016/j.adnc.2006.04.008 16890134

[28] Schellenberg J, Links MG, Hill JE, Hemmingsen SM, Peters GA, Dumonceaux TJ. Pyrosequencing of chaperonin-60 (cpn60) amplicons as a means of determining microbial community composition. Methods Mol Biol. 2011;733(143–58. 10.1007/978-1-61779-089-8_10 21431768

[29] Links MG, Chaban B, Hemmingsen SM, Muirhead K, Hill JE. mPUMA: a computational approach to microbiota analysis by *de novo* assembly of operational taxonomic units based on protein-coding barcode sequences. Microbiome. 2013;1(1): 23 10.1186/2049-2618-1-23 24451012 PMC3971603

[30] Hill JE, Penny SL, Crowell KG, Goh SH, Hemmingsen SM. cpnDB: a chaperonin sequence database. Genome Res. 2004;14(8): 1669–75. 10.1101/gr.2649204 15289485 PMC509277

[31] van Kuppeveld FJ, van der Logt JT, Angulo AF, van Zoest MJ, Quint WG, Niesters HG, et al Genus- and species-specific identification of mycoplasmas by 16S rRNA amplification. Appl Environ Microbiol. 1992;58(8): 2606–15. 1381174 10.1128/aem.58.8.2606-2615.1992 PMC195828

[32] Yoshida T, Maeda S, Deguchi T, Ishiko H. Phylogeny-based rapid identification of mycoplasmas and ureaplasmas from urethritis patients. J Clin Microbiol. 2002;40(1): 105–10. 10.1128/JCM.40.1.105-110.2002 11773101 PMC120092

[33] Teng LJ, Zheng X, Glass JI, Watson HL, Tsai J, Cassell GH. *Ureaplasma urealyticum* biovar specificity and diversity are encoded in multiple-banded antigen gene. J Clin Microbiol. 1994;32(6): 1464–9. 8077390 10.1128/jcm.32.6.1464-1469.1994 PMC264020

[34] Teng LJ, Ho SW, Ho HN, Liaw SJ, Lai HC, Luh KT. Rapid detection and biovar differentiation of *Ureaplasma urealyticum* in clinical specimens by PCR. J Formos Med Assoc. 1995;94(7): 396–400. 7549563

[35] Jensen JS, Borre MB, Dohn B. Detection of *Mycoplasma genitalium* by PCR amplification of the 16S rRNA gene. J Clin Microbiol. 2003;41(1): 261–6. 10.1128/JCM.41.1.261-266.2003 12517858 PMC149599

[36] Caporaso JG, Kuczynski J, Stombaugh J, Bittinger K, Bushman FD, Costello EK, et al QIIME allows analysis of high-throughput community sequencing data. Nat Methods. 2010;7(5): 335–6. 10.1038/nmeth.f.303 20383131 PMC3156573

[37] Oksanen J, Blanchet FG, Kindt R, Legendre P, Minchin PR, O'Hara RB, et al. vegan: Community Ecology Package. R package version 2.0–10. 2012. Available: https://cran.r-project.org/web/packages/vegan.

[38] Bulletins-Obstetrics ACoP. ACOG Practice Bulletin No. 80: premature rupture of membranes. Clinical management guidelines for obstetrician-gynecologists. Obstet Gynecol. 2007;109(4): 1007–19. 10.1097/01.AOG.0000263888.69178.1f 17400872

[39] Albert AY, Chaban B, Wagner EC, Schellenberg JJ, Links MG, van Schalkwyk J, et al A study of the vaginal microbiome in healthy Canadian women utilizing cpn60-based molecular profiling reveals distinct *Gardnerella* subgroup community state types. PLoS One. 2015;10(8): e0135620 10.1371/journal.pone.0135620 26266808 PMC4534464

[40] Gajer P, Brotman RM, Bai G, Sakamoto J, Schutte UM, Zhong X, et al Temporal dynamics of the human vaginal microbiota. Sci Transl Med. 2012;4(132): 132ra52 10.1126/scitranslmed.3003605 22553250 PMC3722878

[41] Chaban B, Links MG, Jayaprakash TP, Wagner EC, Bourque DK, Lohn Z, et al Characterization of the vaginal microbiota of healthy Canadian women through the menstrual cycle. Microbiome. 2014;2(23 10.1186/2049-2618-2-23 25053998 PMC4106219

[42] Martius J, Krohn MA, Hillier SL, Stamm WE, Holmes KK, Eschenbach DA. Relationships of vaginal *Lactobacillus* species, cervical *Chlamydia trachomatis*, and bacterial vaginosis to preterm birth. Obstet Gynecol. 1988;71(1): 89–95. 3336545

[43] Foxman B, Wen A, Srinivasan U, Goldberg D, Marrs CF, Owen J, et al Mycoplasma, bacterial vaginosis-associated bacteria BVAB3, race, and risk of preterm birth in a high-risk cohort. Am J Obstet Gynecol. 2014;210(3): 226 e1-7. 10.1016/j.ajog.2013.10.003 24096128 PMC3943817

[44] Holst E, Goffeng AR, Andersch B. Bacterial vaginosis and vaginal microorganisms in idiopathic premature labor and association with pregnancy outcome. J Clin Microbiol. 1994;32(1): 176–86. 8126176 10.1128/jcm.32.1.176-186.1994 PMC262991

[45] Gravett MG, Nelson HP, DeRouen T, Critchlow C, Eschenbach DA, Holmes KK. Independent associations of bacterial vaginosis and *Chlamydia trachomatis* infection with adverse pregnancy outcome. JAMA. 1986;256(14): 1899–903. 3761496

[46] Joesoef MR, Hillier SL, Wiknjosastro G, Sumampouw H, Linnan M, Norojono W, et al Intravaginal clindamycin treatment for bacterial vaginosis: effects on preterm delivery and low birth weight. Am J Obstet Gynecol. 1995;173(5): 1527–31. 7503196 10.1016/0002-9378(95)90644-4

[47] Menard JP, Fenollar F, Henry M, Bretelle F, Raoult D. Molecular quantification of *Gardnerella vaginalis* and *Atopobium vaginae* loads to predict bacterial vaginosis. Clin Infect Dis. 2008;47(1): 33–43. 10.1086/588661 18513147

[48] Verstraelen H, Verhelst R, Claeys G, De Backer E, Temmerman M, Vaneechoutte M. Longitudinal analysis of the vaginal microflora in pregnancy suggests that *L*. *crispatus* promotes the stability of the normal vaginal microflora and that *L*. *gasseri* and/or *L*. *iners* are more conducive to the occurrence of abnormal vaginal microflora. BMC Microbiol. 2009;9(116 10.1186/1471-2180-9-116 19490622 PMC2698831

[49] Santiago GL, Tency I, Verstraelen H, Verhelst R, Trog M, Temmerman M, et al Longitudinal qPCR study of the dynamics of *L*. *crispatus*, *L*. *iners*, *A*. *vaginae*, (sialidase positive) *G*. *vaginalis*, and *P*. *bivia* in the vagina. PLoS One. 2012;7(9): e45281 10.1371/journal.pone.0045281 23028904 PMC3448655

[50] Jakobsson T, Forsum U. *Lactobacillus iners*: a marker of changes in the vaginal flora? J Clin Microbiol. 2007;45(9): 3145 10.1128/JCM.00558-07 17652481 PMC2045263

[51] Petricevic L, Domig KJ, Nierscher FJ, Sandhofer MJ, Fidesser M, Krondorfer I, et al Characterisation of the vaginal *Lactobacillus* microbiota associated with preterm delivery. Sci Rep. 2014;4(5136 10.1038/srep05136 24875844 PMC4038809

[52] Macklaim JM, Gloor GB, Anukam KC, Cribby S, Reid G. At the crossroads of vaginal health and disease, the genome sequence of *Lactobacillus iners* AB-1. Proc Natl Acad Sci U S A. 2011;108 Suppl 1(4688–95. 21059957 10.1073/pnas.1000086107 PMC3063587

[53] Pybus V, Onderdonk AB. A commensal symbiosis between *Prevotella bivia* and *Peptostreptococcus anaerobius* involves amino acids: potential significance to the pathogenesis of bacterial vaginosis. FEMS Immunol Med Microbiol. 1998;22(4): 317–27. 9879923 10.1111/j.1574-695X.1998.tb01221.x

[54] Pybus V, Onderdonk AB. Evidence for a commensal, symbiotic relationship between *Gardnerella vaginalis* and *Prevotella bivia* involving ammonia: potential significance for bacterial vaginosis. J Infect Dis. 1997;175(2): 406–13. 9203662 10.1093/infdis/175.2.406

[55] Aroutcheva A, Ling Z, Faro S. *Prevotella bivia* as a source of lipopolysaccharide in the vagina. Anaerobe. 2008;14(5): 256–60. 10.1016/j.anaerobe.2008.08.002 18849004 PMC2651005

[56] Zozaya-Hinchliffe M, Martin DH, Ferris MJ. Prevalence and abundance of uncultivated *Megasphaera*-like bacteria in the human vaginal environment. Appl Environ Microbiol. 2008;74(5): 1656–9. 10.1128/AEM.02127-07 18203860 PMC2258643

[57] Datcu R, Gesink D, Mulvad G, Montgomery-Andersen R, Rink E, Koch A, et al Vaginal microbiome in women from Greenland assessed by microscopy and quantitative PCR. BMC Infect Dis. 2013;13(480 10.1186/1471-2334-13-480 24131550 PMC3853076

[58] Gauthier S, Tetu A, Himaya E, Morand M, Chandad F, Rallu F, et al The origin of *Fusobacterium nucleatum* involved in intra-amniotic infection and preterm birth. J Matern Fetal Neonatal Med. 2011;24(11): 1329–32. 10.3109/14767058.2010.550977 21314291

[59] Hill GB. Preterm birth: associations with genital and possibly oral microflora. Ann Periodontol. 1998;3(1): 222–32. 10.1902/annals.1998.3.1.222 9722706

[60] Goldenberg RL, Iams JD, Mercer BM, Meis PJ, Moawad AH, Copper RL, et al The preterm prediction study: the value of new vs standard risk factors in predicting early and all spontaneous preterm births. NICHD MFMU Network. Am J Public Health. 1998;88(2): 233–8. 9491013 10.2105/ajph.88.2.233 PMC1508185

[61] Waites KB, Katz B, Schelonka RL. Mycoplasmas and ureaplasmas as neonatal pathogens. Clin Microbiol Rev. 2005;18(4): 757–89. 10.1128/CMR.18.4.757-789.2005 16223956 PMC1265909

[62] Taylor-Robinson D, Lamont RF. Mycoplasmas in pregnancy. BJOG. 2011;118(2): 164–74. 10.1111/j.1471-0528.2010.02766.x 21091927

[63] Kim MJ, Romero R, Gervasi MT, Kim JS, Yoo W, Lee DC, et al Widespread microbial invasion of the chorioamniotic membranes is a consequence and not a cause of intra-amniotic infection. Lab Invest. 2009;89(8): 924–36. 10.1038/labinvest.2009.49 19506551 PMC2743483

[64] Clark GW, Tillier ER. Loss and gain of GroEL in the Mollicutes. Biochem Cell Biol. 2010;88(2): 185–94. 10.1139/o09-157 20453921

[65] Grattard F, Soleihac B, De Barbeyrac B, Bebear C, Seffert P, Pozzetto B. Epidemiologic and molecular investigations of genital mycoplasmas from women and neonates at delivery. Pediatr Infect Dis J. 1995;14(10): 853–8. 8584311 10.1097/00006454-199510000-00007

[66] Paul VK, Gupta U, Singh M, Nag VL, Takkar D, Bhan MK. Association of genital mycoplasma colonization with low birth weight. Int J Gynaecol Obstet. 1998;63(2): 109–14. 9856315 10.1016/s0020-7292(98)00135-0

[67] Bayraktar MR, Ozerol IH, Gucluer N, Celik O. Prevalence and antibiotic susceptibility of *Mycoplasma hominis* and *Ureaplasma urealyticum* in pregnant women. Int J Infect Dis. 2010;14(2): e90–5. 10.1016/j.ijid.2009.03.020 19515594

[68] Larsen B, Hwang J. *Mycoplasma*, *Ureaplasma*, and adverse pregnancy outcomes: a fresh look. Infect Dis Obstet Gynecol. 2010;2010(10.1155/2010/521921 PMC2913664 20706675

[69] Pepin J, Labbe AC, Khonde N, Deslandes S, Alary M, Dzokoto A, et al *Mycoplasma genitalium*: an organism commonly associated with cervicitis among west African sex workers. Sex Transm Infect. 2005;81(1): 67–72. 10.1136/sti.2003.009100 15681727 PMC1763741

[70] Gaydos C, Maldeis NE, Hardick A, Hardick J, Quinn TC. *Mycoplasma genitalium* as a contributor to the multiple etiologies of cervicitis in women attending sexually transmitted disease clinics. Sex Transm Dis. 2009;36(10): 598–606. 10.1097/OLQ.0b013e3181b01948 19704398 PMC2924808

[71] Cazanave C, Manhart LE, Bebear C. *Mycoplasma genitalium*, an emerging sexually transmitted pathogen. Med Mal Infect. 2012;42(9): 381–92. 10.1016/j.medmal.2012.05.006 22975074

[72] Davidson KM. Detection of premature rupture of the membranes. Clin Obstet Gynecol. 1991;34(4): 715–22. 1778013 10.1097/00003081-199112000-00007

[73] Walther-Antonio MR, Jeraldo P, Berg Miller ME, Yeoman CJ, Nelson KE, Wilson BA, et al Pregnancy's stronghold on the vaginal microbiome. PLoS One. 2014;9(6): e98514 10.1371/journal.pone.0098514 24896831 PMC4045671

[74] Human Microbiome Project C. Structure, function and diversity of the healthy human microbiome. Nature. 2012;486(7402): 207–14. 10.1038/nature11234 22699609 PMC3564958

