# Supplementary figures and images for: High Diversity and Variability in the Vaginal Microbiome in Women following Preterm Premature Rupture of Membranes (PPROM): A Prospective Cohort Study
Source: PLoS One. 2016 Nov 18;11(11):e0166794. doi: 10.1371/journal.pone.0166794 (PMC5115810; doi:10.1371/journal.pone.0166794)

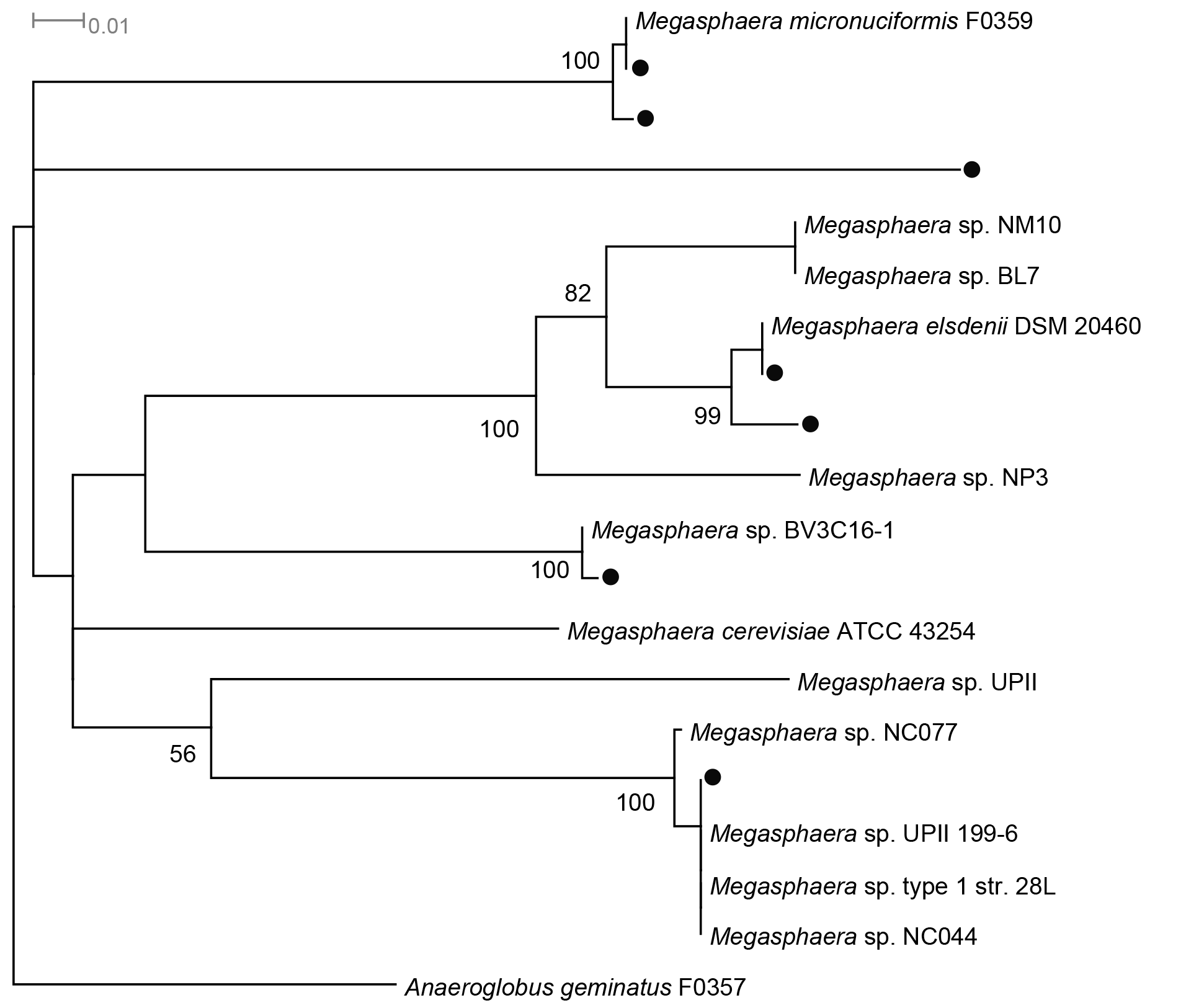

Supplement: S1 Fig — The tree is based on a 315 bp alignment and was constructed using the F84 distance algorithm followed by neighbour joining using the PHYLIP software package (Felsenstein J. PHYLIP—phylogeny inference package (version 3.2). Cladistics. 1989;5: 164–6). Bootstrap values (>50%) are indicated at nodes. (TIF) [file pone.0166794.s001.tif]

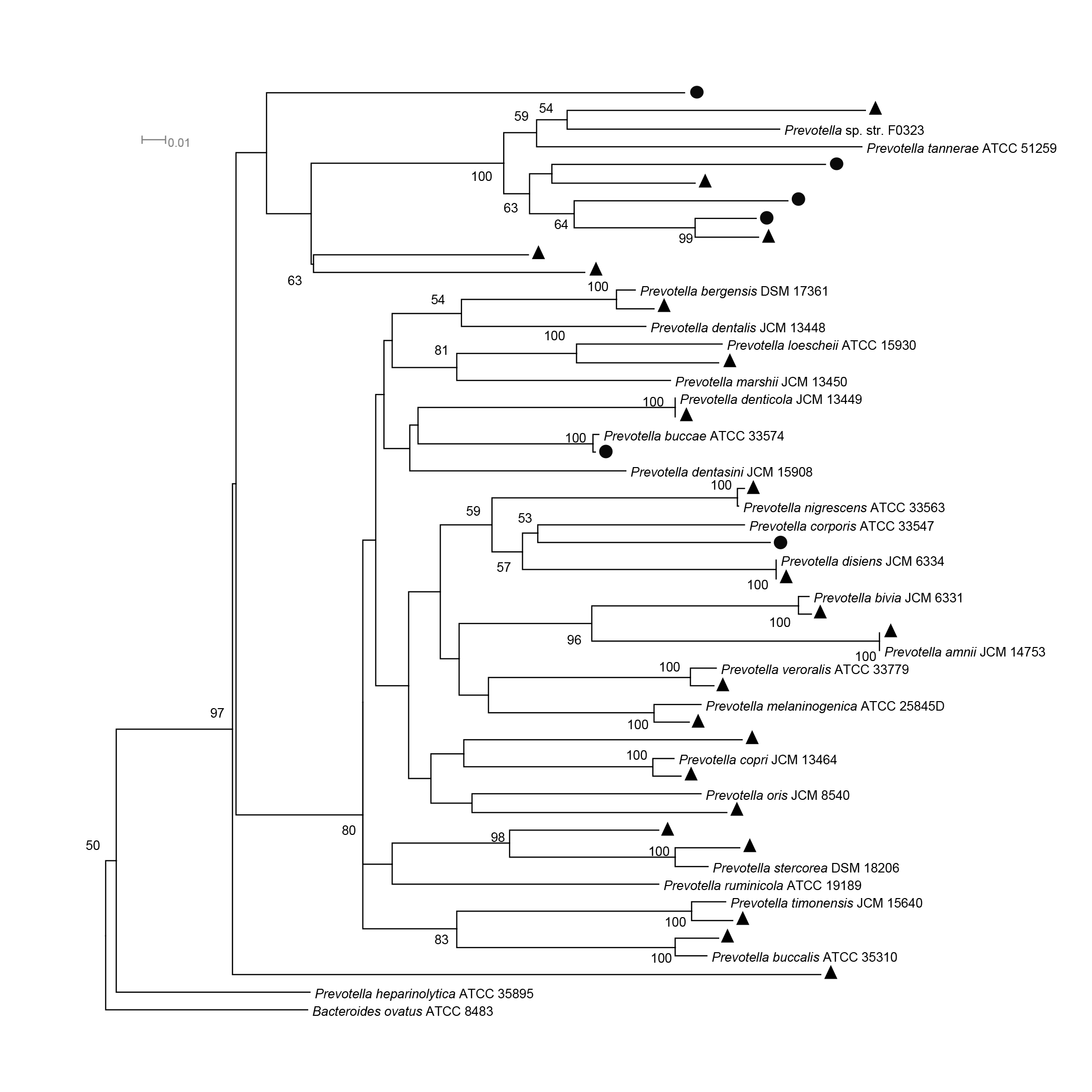

Supplement: S2 Fig — Study sequences detected in T0 samples are indicated by triangles. Sequences represented by circles were detected only in weekly or delivery vaginal samples. The tree is based on a 300 bp alignment and was constructed using the F84 distance algorithm followed by neighbour joining using the PHYLIP software package (Felsenstein J. PHYLIP—phylogeny inference package (version 3.2). Cladistics. 1989;5: 164–6). Only 28 of the 36 identified Prevotella-like OTU could be included in the tree since the remaining sequences did not provide sufficient overlap to be included in the alignment. Bootstrap values (>50%) are indicated at node. (TIF) [file pone.0166794.s002.tif]

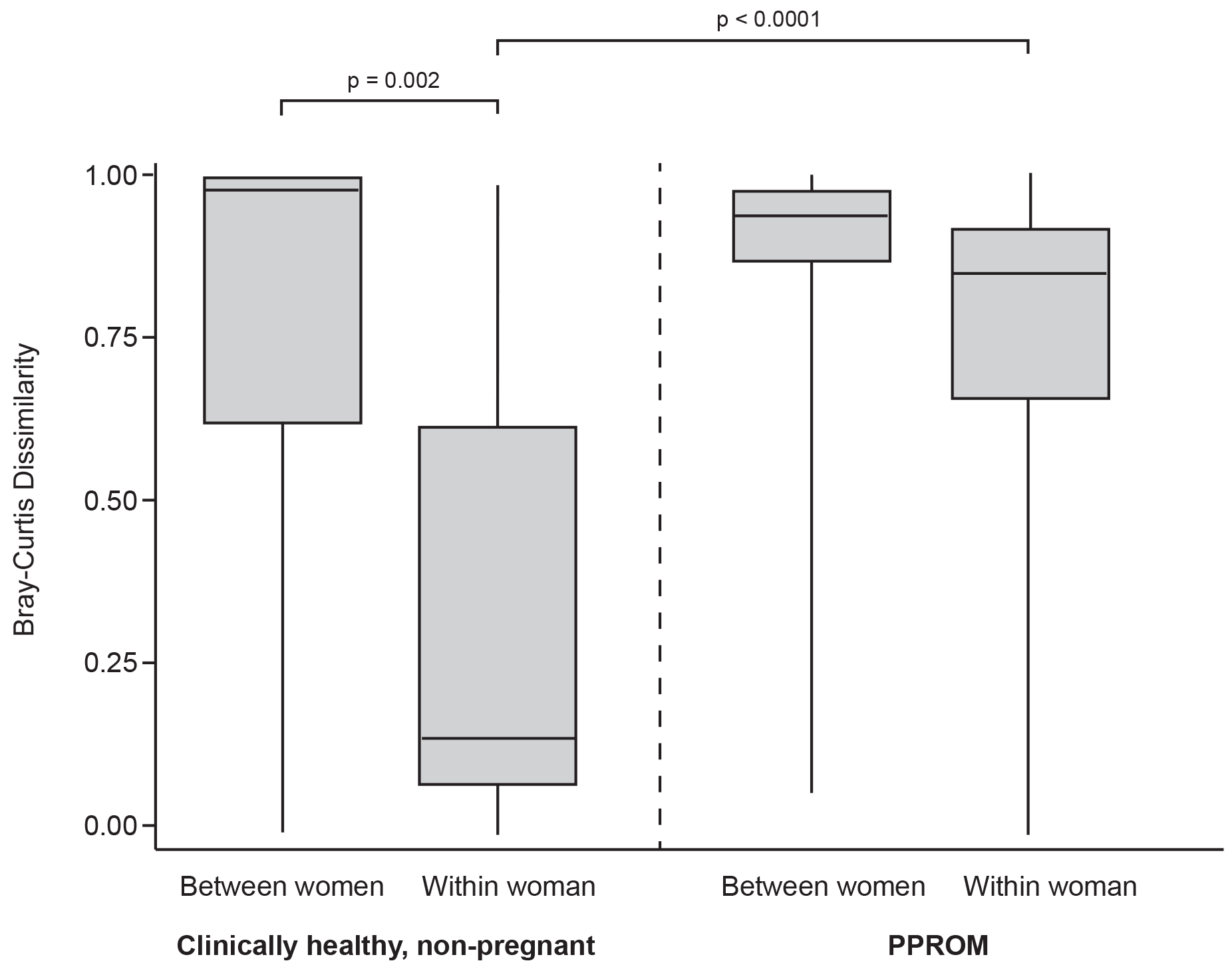

Supplement: S3 Fig — (TIF) [file pone.0166794.s003.tif]
