# Supplementary material for: High Diversity and Variability in the Vaginal Microbiome in Women following Preterm Premature Rupture of Membranes (PPROM): A Prospective Cohort Study
Source: PLoS One. 2016 Nov 18;11(11):e0166794. doi: 10.1371/journal.pone.0166794 (PMC5115810; doi:10.1371/journal.pone.0166794)
Supplement: S1 Table — (DOC) [file pone.0166794.s004.doc]

**S1 Table**. Prevalence of nearest neighbour species accounting for at least 1% of the total sequence reads in the study.

| **Nearest neighbour** | **No. of OTU** | **% identity** | **No. of positive subjects (/36)** | **No. of positive samples (/70)** | **Proportion of data (%)** |
| --- | --- | --- | --- | --- | --- |
| *Megasphaera* sp. UPII 199-6 | 1 | 99.6 | 36 | 70 | 3.0 |
| *Lactobacillus iners* DSM 13335 | 2 | 93.9-99.3 | 36 | 60 | 8.8 |
| *Lactobacillus crispatus* CECT4840 | 2 | 95.3-99.8 | 35 | 62 | 5.8 |
| *Prevotella timonensis* JCM 15640 | 1 | 97.1 | 34 | 64 | 12.4 |
| *Gardnerella vaginalis* 409-05 (Group A) | 2 | 88.8-99.5 | 31 | 51 | 11.3 |
| *Escherichia coli* KTE143 | 1 | 99.1 | 31 | 50 | 1.8 |
| *Lactobacillus jensenii* ATCC 25258 | 2 | 98.9-99.2 | 30 | 43 | 4.2 |
| *Corynebacterium accolens* ATCC 49725 | 4 | 91.2 | 29 | 59 | 2.6 |
| *Gardnerella vaginalis* ATCC 14018 (Group C) | 1 | 99.5 | 29 | 48 | 3.0 |
| *Streptococcus pseudopneumoniae* ATCC BAA-960 | 3 | 94.2-94.4 | 29 | 45 | 2.6 |
| *Alloscardovia omnicolens* LMG 23791 | 1 | 97.3 | 27 | 46 | 3.6 |
| BVAB2 | 2 | 99.6-99.8 | 27 | 38 | 1.38 |
| *Prevotella bivia* JCM 6331 | 1 | 99.1 | 25 | 50 | 2.6 |
| *Peptoniphilus harei* ACS-146-V-Sch2b | 4 | 73.6-98.6 | 25 | 45 | 1.3 |
| *Corynebacterium striatum* ATCC 6940 | 1 | 94 | 22 | 47 | 1.4 |
| *Bifidobacterium infantis* 6w-50 | 1 | 99.3 | 22 | 35 | 2.5 |
| *Actinomyces* sp. | 4 | 74.1-100 | 21 | 36 | 2.7 |
| *Streptococcus oralis* ATCC 35037 | 2 | 88.7-93.8 | 17 | 25 | 1.0 |
| *Prevotella veroralis* ATCC 33779 | 1 | 98.2 | 15 | 27 | 3.1 |
| *Streptococcus agalactiae* ATCC 13813 | 1 | 98.7 | 12 | 22 | 1.8 |
